# Challenges and benefits: Volunteerism among older adults during Covid-19

**DOI:** 10.1093/geroni/igab046.3363

**Published:** 2021-12-17

**Authors:** Alicia Sellon, Tina K Newsham, Renee DuMont, Claire Hollifield, Alicia Thomas

**Affiliations:** 1 University of North Carolina Wilmington, Wilmington, North Carolina, United States; 2 University of North Carolina Wilmington, University of North Carolina Wilmington, North Carolina, United States; 3 University of North Carolina Wilmington, wilmington, North Carolina, United States

## Abstract

Social distancing restrictions and regulations, put in place to reduce the spread of COVID-19, disrupted the daily lives of active older adult volunteers. One year into the pandemic, we used a mixed-methods approach to explore how these regulations had impacted the quality of life, loneliness, and volunteer behavior of 26 older adults who were active volunteers (i.e., at least an hour a week) prior to the start of the pandemic. All the participants were white and non-Hispanic, and the majority were female (65.4%). The average age was 71, with a range from 53 to 87 years old. On average, participant scores on the UCLA loneliness scale (4.23 ±1.39) indicated a low amount of loneliness and high scores on the Brunnsviken Brief Quality of Life (BBQ) scale (83.54 ±10.97) indicated a high quality of life. Thematic findings from the interviews conveyed that, despite the challenges and risks associated with volunteering during a pandemic, participants valued volunteer work enough to make adjustments or seek out new volunteer activities. The research team identified two overarching themes related to participants' discussions of volunteering during the pandemic: Challenges and changes and Benefits of volunteering during a pandemic. Participants' discussions of how volunteer work changed and why they continued to or sought out new volunteer activities during a pandemic can guide organizations seeking to support or recruit older volunteers, particularly as the pandemic continues. These findings also provide further evidence of the important role that volunteerism can play in the well-being of older adults.

